# Supplementary material for: Genomic structural variation-mediated allelic suppression causes hybrid male sterility in rice
Source: Nat Commun. 2017 Nov 3;8:1310. doi: 10.1038/s41467-017-01400-y (PMC5670240; doi:10.1038/s41467-017-01400-y)
Supplement: Supplementary file 1 — Supplementary Information [file 41467_2017_1400_MOESM1_ESM.pdf]

Supplementary Figures 1-10

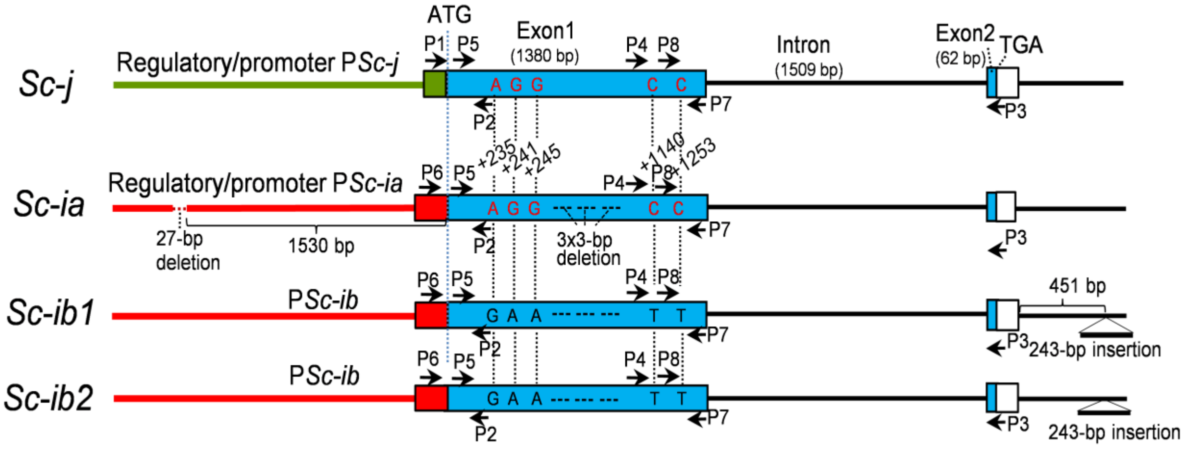

Supplementary Figure 1. Structural variations among *Sc-j* and *Sc-i* paralogs. *Sc-ia* and *Sc-ib1/ib2* have a regulatory/promoter/5'-UTR sequence different from that of *Sc-j*. Only a subset of the nucleotide variations and the primers used are shown (*Sc-ib1* and *Sc-ib2* have identical sequence). All the nucleotide and amino acid variations and the primers used are indicated in Supplementary Figs. 2-4 and Supplementary Table 3.

Supplementary Fig. 2(a)

```
Sc-j    ATGGCGCCATCAGGTTCGTTGACATGAAGAGGACACACGGTCGCTGAGGATTTGAAGCTGGCCATCTGGATCCTGTCGCT   -2217
Sc-is   ATGGCGCCATCAGGTTCGTTGACATGAAGAGGACACACGGTCGCTGAGGATTTGAAGCTGGCCATCTGGATCCTGTCGCT   -2212
Sc-ia   TGCATATTTATATACATTCTAAAGAGCCCTGAGTAGCATCTTTTGTATGCTCTAACTCTCCAACCTTCCACACAGAAGTA   -2218

Sc-j    GGACTCCGGCGGCGAGTGGAGGAAAGGCCCGGAGTGTCGTGTCGGAGACATTTGGGCAAGCGAAAAGTACGTTGTCATGG   -2037
Sc-is   GGACTCCGGCGGCGAGTGGAGGAAAGGCCCGGAGTGTCGTGTCGGAGACATTTGGGCAAGCGAAAAGTACGTTGTCATGG   -2032
Sc-ia   CATAACTACTACAAAAATGAACAAAAACCACCAGAAGGGGTCAAGTGGCAGGCCCTTCCCGAAACAAAGTCCTGTATGTA   -2038

Sc-j    GAGTGCCGCGGATTAGTCCGCCTCCGGTGTGCCCTGTCTTGCGCACGGTCAACGATAATGTCGTCTGCGTTATCGTGAAC   -1957
Sc-is   GAGTGCCGCGGATTAGTCCGCCTCCGGTGTGCCCTGTCTTGCGCACGGTCAACGATAATGTCGTCTGCGTTATCGTGAAC   -1952
Sc-ia   TCAAACCAAACCCTAACTACCAAAGCAAATGAAAGACAGACCATATGCATAGTCTACTATTAAAAATTGGGTTCATGGAA   -1958

Sc-j    GATGCCGACGAGACTGTGTACGGGAGTGTGGATGTCATAGCCCAGCATTTTCTTGCCATCGACGTGGAACGTAAGGATCG   -1877
Sc-is   GATGCCGACGAGACTGTGTACGGGAGTGTGGATGTCATAGCCCAGCATTTTCTTGCCATCGACGTGGAACGTAAGGATCG   -1872
Sc-ia   CATAGTAACAACATGCCTTCCTCTGACTAATATTTTATATGTGTAACCTGTATCTGTTCAGAGTTATGATAGCATTATTT   -1878

Sc-j    AGGTGGGTGCTCTGCTGTCCGTCGTTACCAAGCTTCACGCCTTCACCCTGATGGTGGGGTAGAACCTGTACGGCTGTACC   -1797
Sc-is   AGGTGGGTGCTCTGCTGTCCGTCGTTACCAAGCTTCACGCCTTCACCCTGATGGTGGGGTAGAACCTGTACGGCTGTACC   -1792
Sc-ia   GTTTGGGGAAACCAGATAGTTTGCCACACTCCTTGGGTGGCACATGATGATTTGCCACCCCCTCCATCCATGACATGTCA   -1798

Sc-j    CTAACCACATGGCTTGCGAATTCAGTGAGTACATGGAACACTGACAGGTATAGAGGTACAAATAATAGCCTCTAGTACTG   -1717
Sc-is   CTAACCACATGGCTTGCGAATTCAGTGAGTACATGGAACACTGACAGGTATAGAGGTACAAATAATAGCCTCTAGTACTG   -1712
Sc-ia   CACTCACACACATGGTTGGAAAATTAACATGAAGGATGAATTTATTTTCTTTTTTTAAGAGTAAATTGCGATTAACCCAA   -1718

Sc-j    CTAGGACCATCCAAAATACTTGTCATTGTCAATTGTTGAGTAAATTTTATTTTAGGGAACT----TTTATTATCAATGTT   -1641
Sc-is   CTAGGACCATCCAAAATACTTGTCATTGTCAATTGTTGAGTAAATTTTATTTTAGGGAACTTTTATTTATTATCAATGTT   -1632
Sc-ia   GTTTGTACCTAAAGCAATTAAAGGGTTTGAACATAAACAGTACTGCTGGTATGCCAGCTTTTACAGGAAACAGCATGACC   -1638

Sc-j    TCATTTTGTACCATCCTTTAACCAATGATTTTACTTTAAATCGGGTATTTTTTTTACCTTTGTTTTACATTGGTTCACCC   -1561
Sc-is   TCATTTTGTACCATCCTTTAACCAATGATTTTACTTTAAATCGGGTA--TTTTTTACCTTTGTTTTACATTGGTTCACCC   -1554
Sc-ia   AAACTAGCTGCAAGACATTCCTAGCAGGTTCATAACCCAACAAATGCCATATTGCCACCGTAACGCATCATTTCACAGTC   -1558

Sc-j    TAACTCTCTTCTCAAGTATAAGTATATAAATGATCTTAAGTGCGTTGGCTCCTACTCATCAATAAACTCACATAGCAATA   -1481
Sc-is   TAACTCTCTTCTCAAGTATAAGTATATAAATGATCTTAAGTGCGTTGGCTTCTACTCATCAATAAACTCACATAGCAATA   -1474
Sc-ia   AAGTCAAATATTGACACAGATGTAGATATCCAAGATTGTAATATGTGAATACAAAACCATGTACGTGAAGGATAATTTTC   -1478

Sc-j    TGCAAGATAATTTCTTTGGCATATAACAAGTTTGATGTAGATGGATAAAATAGTTGAGGGGTTCAAAATATCACAAAAGT   -1401
Sc-is   TGCAAGATAATTTCTTTGGCATATAACAAGTTTGATGTAGATGGATAAAATAGTTGAGGGGTTCAAAATATCACAAAAAT   -1394
Sc-ia   ACAAAATTATAAACATGAATCATGAGCTAAAAAGTTTAAATATGGACAAAAAAAAACCCTGAATTGATAAAACAGTCCAT   -1398

Sc-j    AAAAATACCCGATCCAAAGTAACAACATTGTTTAAAGGGTGATCTAAGATGAAATATCGGTAATAAAAGATGACAAAAAG   -1301
Sc-is   AAAAATACCCGATCCAAAGTAACAACATTGTTTAAAGGGTGGTCTAAGATGAAATATCGGTAATAAAAGATGACAAAAAG   -1294
Sc-ia   TCTTGATACTGGTGTTCGTGAGATCGAAAGCATACACAGGATATACCTTACACATGGCTTCAACCCTCCTTTTATATAGG   -1298

Sc-j    TAAAATTTACTCTTAATTTTTCATGCATATTTAGTTCTAAAGTAAAAATCTGTATATTATGTGGTGTAATTAGGATTTAT   -1241
Sc-is   TAAAATTTACTCTTAATTTTTCATGCATATTTAGTTCTAAAGTAAAAATCTGTATATTATGTGGTGTAATTAGGATTTAT   -1234
Sc-ia   GCTGCGTTTAGTTCCCCGTCAAAATTGGAAGTTTGAAGAAATCGGAACGATGTGATGGAAAAGTTGGAAGTTTGTGTATA   -1238

Sc-j    ATGAAAGGTGATTTATCTACATGTAATAGGTCAAATCAATGGTGGTCCACTATACAATTTTTATCATGTTATGTATTATA   -1161
Sc-is   ATGAAAGGTGATTTATCTACGTGTAATAGGTCAAATCAATGGTGGTCCACTATACAATTTTTATCATGTTATGTATTATA   -1154
Sc-ia   GGAAAGTTTGATGTGACGGAAAAGTTGGAAGTTTGAAGAAAAAAAATTAGAATCTAAATAGGGCCTAGGTTGAGAGATGA   -1158

Sc-j    TTTAGAAATAAGACTACTTCACCCCTAAAGTATTGATGCTGATCTACATAACCCCTGGAGTATGAAACTGGGTATTTTAT   -1081
Sc-is   TTTAGGAATAAGACTACTTCACCCCTAAAGTATTGATGCTGATCTACATAA-CCCTGGAGTATGAAACTGGGTATTTTAT   -1075
Sc-ia   GTCTGAGCATCAATAAAATTGGCATCCCGGCGAAATGTTCGAGCGCACCGCCAAAAGTGTTAAATTATCAAATTTCTCCC   -1078

Sc-j    TCCGTAAAGTATCAAAATCAGTTCAAATAACACCTAGAGTGGTTATTGAAACGGGCTCTTGAACTAAAGAGGAGACTTTG   -1001
Sc-is   TCCGTAAAGTGTCAAAATCAGTTCAAATAACACCTAGAGTGGTTATTGAAACGGGCTCTTGAACTAAAGAGGAGACTTTG   -995
Sc-ia   AAAAACAAGCGCGTGAGATAACAACAGGATTAAAGAAAGAAAATTTCAACATGAAGTCGACAAAAACCGGAAACATCTGT   -998
```

(continued)

Supplementary Fig. 2(b)

```
Sc-j     AAATGGGCTAAGTATTTTATTCACAAGTGAAACGCCCGAGACTAATGTAAATTAGTGTTAGTATAGTAACTTTAGGCTTG   -921
Sc-is    AAATGGGCTAAGTATTTTATTCACAAGTGAAACGCCCGAGACTGATGTAAATTAGTGTTAGTATAGTAACTCTAGGCTTG   -915
Sc-ia    TGACTAGTGTTGTAGATCCAGTATAGCAAAAGACCAAGAATTAAAACAACGGCTTAAAAACAGCACAAACAGAATGCTAA   -918

Sc-j     TCGATTAGTGCGTGAAAAGCAATAGTTAGATAAATTTGGGTTTACTAGACTAATCTTTGCGCCACACAATCATTTGCATG   -841
Sc-is    TCGATTAGTGCGTGAAAAGCAATAGTTAGATAAATTTGGGTTTACTAGACTAATCTTTGCGCCACACAATCATTTACATG   -835
Sc-ia    AAGGTTGCAAACCAGTAAGCAAGTATAATGGACGACACATTTTGTTCGGATCATCACCTCTTTCTCCTTATCCATCAGAA   -838

Sc-j     TCAAAACTACCTATAAAACCACTGTAGGGGTTATTTGAACCGATTTTCATACTTCAAAGGTAGAAATCTAGTTTCGTACT   -761
Sc-is    TCAAAACTACCTATAAAACCACTGTAGGGGTTATTTGAACCGATTTTCATACTTCAAAGGTAGAAATCTAGTTTCGTACT   -755
Sc-ia    AACACCGAGACAGCGAAAGGTCGACGAGCCCATCCTTGGAAGTGGACGCCAACTCCGTAGCCGCCACTGCAGCCTCGTGC   -758

Sc-j     TTATAAGTGGTTAAATAGACCAACCCCAACACTTCAGGGTATTCCTTATATCTACTATAGTTTTAACTTTGAGTTAAATC   -681
Sc-is    TTATAAGTGGTTAAATAGACCAACCCCAACACTTCAGGGTATTCCTTATATCTACTATAGTTTTAACTTTGAGTTAAATC   -675
Sc-ia    GAGGCAGCCGCTGATGTAGGCGCCGGCGGCACTTGATCGTCCACGATCAGCTTCTGCCTCAGATCTTTCTTCTCCAGCGT   -678

Sc-j     TTAAGGTGGTCCATGCCCTACGGCAGTAGCGGAGCCAGAAATTTAAATCTGTGGGGTCAATTACTAGTGGTTTGTATTTA   -601
Sc-is    TTAAGGTGGTCCATGCCCCACGGCAGTAGCGGAGCCAGAAATTTAAACCTGTGGGGTCAATTACTAGTGGTTTGTATTTA   -595
Sc-ia    TGCTGCCGGTTCCAACGACGATGGAACCGCCATGGGAGCTAGTAAGGACACCACCATCGGACGCGAATCAGAACAGCGGC   -598

Sc-j     AGTGGGATCATTTCTCTTTATTGTTTTGGATTTATACATAAAAATTGAGGTTTATAGGACGTTTTTTAAAAAGCTGGGGT   -521
Sc-is    AGTGGAGTCATTTCTCTTTATTGTTTTGGATTTATACATAAAAATTGAGGTTTATAGGACGTTTTTCAAAAAGTTGGGGT   -515
Sc-ia    AGCCGGCGCTTGACGTGCCGCGCCCGTGAGCACCGAGCCCGTCGGCGTCGCCTCGGTCCCTGCCGCGGCAGCGGCTGCGG   -518

Sc-j     CGGCCGACCCCACAGAAATGGCCTAAATCCACCACTGCCCCAGGTGCCACCTAGTTAACTCTGCATATGGGCCTATGATC   -441
Sc-is    CGGCCGACCCTACAGAAATGGCCTAAATCCACCACTGCCCCAGGTGCCACCTAGTTAACTCTGCATATGGGCCTATGATC   -435
Sc-ia    CTGCGGCGTCGACGGATCATTAGGGTGCGGAAGGAGTGAGAGAGCGGACAGCGGAGGAACAAGGTGCTGTCTCTCACCCT   -438

Sc-j     CCTTCGATCACTTTTCATTACTACAATGGTGTTTGAAACTGATGGATATTAAGCTGGTGCATGTAGAATGAAGAAAATAA   -361
Sc-is    CCTTCGATCACTTTTCATA--TACAATGGTGTTTGAAACTGATGGATATTAAGCTGGTGCATGTAGAATGAAGAAAATAA   -357
Sc-ia    TCCTTCCACTGCACCTATAGCCTTTTCATCTCCTGCACCTATGCCTAGTCCACAAGACAGAGGCGTCCTCCTCTCCTCTC   -358

Sc-j     TTAACACATTATTGATTAAAATTTAATTATTAAAAACTTGACAAATTAATTTATTTGATAATTTTAATGTAACTTTTATA   -281
Sc-is    TTAAC----GATTGATTAAAATTTAATTATTAAAAACTTGACAAATTAATTTATTTGATAATTTTAATGTAACTTTTATA   -281
Sc-ia    TCTCCTCTCTCCTCTCACCGGTGCATAATGAACCTAGTCTACAAACTAGGCTCCCTTTGTGTTTGTTGCGGGTGGCATAT   -278

Sc-j     TAGAAAGTTTGAAAAGTGTAATAACGGAAACTGAGTTAAAATCTGTACGTGTTAAATATATTATTCCCCTTTTTAAATTT   -201
Sc-is    TAGAAAGTTTGAAAAGTGTAATAACGGAAACTGAGTTAAAATCTGTACGTGTTAAATATATTATTCCCCTTTTTAAATTT   -201
Sc-ia    CCACCGTCCATTAGCCTCTCTATCTCCGCCGCCATATCCACCGTCCATTAGTCTCCCGATCCCCGCCACTCTACCTTTGA   -198

Sc-j     ATAGAAGAAGTTAAATATGAACGTTGGAAAACGCTGAACAAGTGAACATATCCCGCGATCCGCATTTGTTGCGACTCGTT   -121
Sc-is    ATAGAAGAAGTTAAATATGAACGTTGGAAAACGCTGAACAAGTGAACATATCCCGCGATCCGCATTTGTTGCGACTCGTT   -121
Sc-ia    TGGGGCCCGTTTCCGATCTGGACTCGTCTTCTCCCTCTCGACGGCACAGTGGGATCTGGGAAGAGGGAAGGGTTTGCTCG   -118

Sc-j     TCGATCAACTGATTCTCCTTCCCGGCCGGACATCCACATCATCCTGCGATCGTTCGTTTCCCTAGTCGTTCCAGGCTTTT   -41
Sc-is    TCGATCAACTGATTCTCCTTCCCGGCCGGACATCCACATCATCCTGCGATCGTTCGTTTCCCTAGTCGTTCCAGGCTTTT   -41
Sc-ia    ATCCTTTGTTCTTGCCGTCTCTTGCACTGATCTAGCTAATTCTTTCTGCTGGGGCACTCGAAAAAAAACTCTTCGACTGC   -38
                                                Start codon
Sc-j     TCAGCTCTACTCCCCTCCACCCAGGCGCAAATCATCCGCCATGGCTCCCGCCTGGGTCTTGCTCGATCGCGTCGTCAAGC   40
Sc-is    TCAGCTCTACTCCCCTCCACCCAGGCGCAAATCATCCGCCATGGCTCCCGCCTGGGTCTTGCTC--TCGTGTCGTCAAGC   38
Sc-ia    TCCCCGTACTCCAAACCCACCCACGCGCAA---ATCCGCGATGGCTCCCACCTGGGTCTTGCTCGATCGCTTCGTCAAGC   40

Sc-j     CCGCCGTCTTCGACGAAGAAGAGAGCAAAGGTAAGGGAGAATCGACCGGCGCCCCAGTGAAGTACCTGCCAGCCAGATTA   80
Sc-is    CCGCCGTCTTCGACGAAGAAGAGAGCAAAGGTAAGGGAGAATCGACCGGCGCCCCAGTGAAGTACCTGCCAGCCAGATTA   78
Sc-ia    CCACTATCTTCGACGAAGAAGAGAGCAAAGGTAAGGGAGAATCGACCGGCGCCCCAGTGAAGTACCTTCCAGCCAGATTA   80

Sc-j     AGGCAGGAAGTCCCCGCCGGCATGCGGGACGTGAAGCCCTACCCAGAGGTTGCGGATCCTCCCATTATATCTCGCTTCTC   120
Sc-is    AGGCAGGAAGGGCCTGTTTGGTTGCTACCCTGAGCAAATAAGCCTGGCCTAAGGTCATGCCTGTAACTCGCCTGGCATCT   118
Sc-ia    AGGCAGGAAGTCCCCGCCGGCATGCGGGACGTGAAGCCCTACCCAGAGGTTGCGGATCCTCCCATTGTATCTCGCTTCTC   120
                   Transposon (T1) insertion            Premature stop codon
```

Supplementary Figure 2. Alignment of the promoter sequences of *Sc* alleles. The regulatory/promoter sequences were determined by sequencing the genomic DNAs of T65, E5 and 9311 (and the public sequence of Nip). "-" shows base deletion. The regulatory/promoter sequences of *Sc-a* and *Sc-ib1/b2* are the same except for a 27-bp insertion/deletion (see Supplementary Fig. 1). A part of the transposon insertion (T1) sequence (in pink) in *Sc-is* is shown. The 2-bp deletion (--) in the exon 1 of *Sc-is* causes a frameshift and produces a stop codon within the transposon insertion sequence (pink).

Supplementary Fig. 3(a)

```
Sc-j         AAAACGCTGAACAAGTGAACATATCCCGCGATCCGCATTTGTTGCGACTCGTTTCGATCAACTGATTCTCCTTCCCGGCC  -94
Sc-ia        TTCTCCCTCTCGACGGCACAGTGGGATCTGGGAAGAGGGAAGGGTTTGCTCGATCCCTTTGTTCTTGCCGTCTCTTGCAC  -91
Sc-ib1/ib2   TTCTCCCTCTCGACGGCACAGTGGGATCTGGGAAGAGGGAAGGGTTTGCTCGATCCCTTTGTTCTTGCCGTCTCTTGCAC  -91
Putative transcription initiation site                                           P6 (for Sc-i)

Sc-j         GGACATCCACATCATCCTGCGATCGTTCGTTTCCCTAGTCGTTCCAGGCTTTTTCAGCTCTACTCCCCTCCACCCAGGCG  -14
Sc-ia        TGATCTAGCTAATTCTTTCTGCTGGGGCACTCGAAAAAAAACTCTTCGACTGCTCCCCGTACTCCAAACCCACCCACGCG  -11
Sc-ib1/ib2   TGATCTAGCTAATTCTTTCTGCTGGGGCACTCGAAAAAAAACTCTTCGACTGCTCCCCGTACTCCAAACCCACCCACGCG  -11
               P1 (for Sc-j)  Start codon
Sc-j         CAAATCATCCGCCATGGCTCCCGCCTGGGTCTTGCTCGATCGCGTCGTCAAGCCCGCCGTCTTCGACGAAGAAGAGAGCA  67
Sc-ia        CAA---ATCCGCGATGGCTCCCACCTGGGTCTTGCTCGATCGCTTCGTCAAGCCCACTATCTTCGACGAAGAAGAGAGCA  67
Sc-ib1/ib2   CAA---ATCCGCGATGGCTCCCACCTGGGTCTTGCTCGATCGCTTCGTCAAGCCCACTATCTTCGACGAAGAAGAGAGCA  67
                   P5
Sc-j         AAGGTAAGGGAGAATCGACCGGCGCCCCAGTGAAGTACCTGCCAGCCAGATTAAGGCAGGAAGTCCCCGCCGGCATGCGG  147
Sc-ia        AAGGTAAGGGAGAATCGACCGGCGCCCCAGTGAAGTACCTTCCAGCCAGATTAAGGCAGGAAGTCCCCGCCGGCATGCGG  147
Sc-ib1/ib2   AAGGTAAGGGAGAATCGACCGGCGCCCCAGTGAAGTACCTTCCAGCCAGATTAAGGCAGGAAGTCCCCGCCGGCATGCGG  147
                              P2                                     P9a (for un-edited Sc-ia)
Sc-j         GACGTGAAGCCCTACCCAGAGGTTGCGGATCCTCCCATTATATCTCGCTTCTCCATGCTGATTTCGCGGAAGGCGATCAG  227
Sc-ia        GACGTGAAGCCCTACCCAGAGGTTGCGGATCCTCCCATTGTATCTCGCTTCTCCATGCTGATTTCGCGGAAGGCGATCAG  227
Sc-ib1/ib2   GACGTGAAGCCCTACCCAGAGGTTGCGGATCCTCCCATTGTATCTCGCTTCTCCATGCTGATTTCGCGGAAGGCGATCAG  227

Sc-j         GGTAGTGAAATCCGTCCGTGTCCGGTGCGCCGACAAGAGCCTTGTCTTGTTCTACGCCGGCACCGGCTTCCCCGGCTTCT  307
Sc-ia        GGTAGTGAAATCCGTCCGTGTCGAGTGCGCCGACAAGAGCCTTGTCTTGTTCTACGCCGGCACCGGCTTCCCCGGCTTC-  306
Sc-ib1/ib2   GGTAGTGGAATCCATCCATGTCGAGTGCGCCGACAAGAGCCTTGTCTTGTTCTACGCCGGCACCGGCTTCCCCGGCTTC-  306

Sc-j         CGTCCCACGGCTGCCACCTGATCTACGACGCCATCGACGGCTCTCTCACTGCGGTGCACACATTCCCCTTTCCTGTGTCC  387
Sc-ia        --TCCCACGGCTGCCACCTGATCTACGACGCCATCGACGGTTCTCTCACTGCAGTGCACACGTTCCCCTTTCCTGTGTCC  384
Sc-ib1/ib2   --TCCCACGGCTGCCACCTGATCTACGACGCCATCGACGGTTCTCTCACTGCAGTGCACACGTTCCCCTTTCCTGTGTCC  384
                                                          P10 (for Sc-i)
Sc-j         GGAGTCGTCTGGGTCGGCAGAGCCGCTGTCCTGCGCCATGCCGGCGGCGGAGGAGGAGGAGGAGACGGTACTACCGCCTC  467
Sc-ia        GGAGTCGTCTGGGTCGGCACAGCCGCTGTCCTGCGCCATGCCGGCGGCGGC---GGAGGAGGAGACGGTACTACCG---C  458
Sc-ib1/ib2   GGAGTCGTCTGGGTCGGCACAGCCGCTGTCCTGCGCCATGCCGGCGGCGGC---GGAGGAGGAGACGGTACTACCG---C  458

Sc-j         CTATGTCATCGCCGAGCTGCTCAGGCCGTTTCATGGCTCCCTTCCCGATGCCACGCTCGTGATGTGGTTGTCGAATTCCC  547
Sc-ia        CTATGTCATCGCCGAGCTGCTCAGGCCGTTCCATGGCTCCCTTCCCGATGCCACGCTCGTGATGTGGTTGTCGAATTCCC  538
Sc-ib1/ib2   CTATGTCATCGCCGAGCTGCTCAGGCCGTTCCATGGCTCCCTTCCCGATGCCACGCTCGTGATGTGGTTGTCGAATTCCC  538

Sc-j         CCGCGTCGACCTCCGGCAGCAACGGCCAATGGGTGAAGGAGGACGTTCGCCTTCCCGGCGAGGTGTGCACGGGCACCGAC  627
Sc-ia        CCGCGTCGACCTCCGGCAGCAATGGCCAATGGGTGAAGGAGGACGTTCGCCTTCCCGGCGAGGTGTGCACGGGCACCGAC  618
Sc-ib1/ib2   CCGCGTCGACCTCCGGCAGCAATGGCCAATGGGTGAAGGAGGACGTTCGCCTTCCCGGCGAGGTGTGCACGGGCACCGAC  618

Sc-j         CCCTTCACCACCGACTTGGTGTTCTCGTTCGGTGAATCGTGCCTGTGCTGGGCCGATCTGTTCATGGGCATCCTGTTCTG  707
Sc-ia        CCCTTCACCACCGACTTGGTGTTCTCGTTCGGTGAATCGTGCCTGTGCTGGGCCGATCTGTTCATGGGCATCCTGTTCTG  698
Sc-ib1/ib2   CCCTTCACCACCGACTTGGTGTTCTCGTTCGGTGAATCGTGCCTGTGCTGGGCCGATCTGTTCATGGGCATCCTGTTCTG  698
                                                  P4
Sc-j         CGACCTTGCGACGCTGCGTGCACCTCGGTTCCGTTTCATCCCATTGCCCAAGGCTTGCTCCTTCGACCCCGTCGGCAAGT  787
Sc-ia        CGACCTTGCGACGCTGCGTGCACCTCGGTTCCGTTTCATCCCATTGCCCAAGGCTTGCTCCTTCGACCCCGTCGGCAAGT  778
Scib1/ib2    CGACCTTGCGACGCTGCGTGCACCTCGGTTCCGTTTCATCCCATTGCCCAAGGCTTGCTCCTTCGACCCCGTCGGCAAGT  778

Sc-j         ATGGCCGGCCTCACATGCCCGAGTTCCGTTCCATGGGCCGCGTCAACGGTGTCATCAGGTTGATCGACATGGAGGGTTTT  867
Sc-ia        ATGGCCGGCCTCACATGCCCGAGTTCCGTTCCATGGGCCGCGTCAACGGTGTCATCAGGTTGATCGACATGGAGGGTTTT  858
Sc-ib1/ib2   ATGGCCGGCCTCACATGCCCGAGTTCCGTTCCATGGGCCGCGTCAACGGTGTCATCAGGTTGATCGACATGGAGGGTTTT  858

Sc-j         ACCAACGAGTACCTGGCCGTGGATGAGGTGAAGCTGACCATCTGGACCCTGTCAGCCGACCTCAGCGAGTGGGAGAAAGG  947
Sc-ia        ACCAACGAGTACCTGGCCGTGGATGAGGTGAAGCTGACCATCTGGACCCTGTCAGCCGACCTCAGCGAGTGGGAGAAAGG  938
Sc-ib1/ib2   ACCAACGAGTACCTGGCCGTGGATGAGGTGAAGCTGACCATCTGGACCCTGTCAGCCGACCTCAGCGAGTGGGAGAAAGG  938
```

(continued)

Supplementary Fig. 3(b)

```
                                                  SpR
Sc-j        CCCGGTGTGTACTGTAGGAGACATTTGGGCCAGTGAGGAATTCGTCGCCATGGGGTTGCCGCAGCTTAGACCGATGTGCC   1027
Sc-ia       CCCGGTGTGTACTGTAGGAGACATTTGGGCCAGTGAGGAATTCGTCGCCATGGGGTTGCCGCAGCTTAGACCGATGTGCC   1018
Sc-ib1/ib2  CCCGGTGTGTACTGTAGGAGACATTTGGGCCAGTGAGGAATTCGTCGCCATGGGGTTGCCGCAGCTTAGACCGATGTGCC   1018

Sc-j        CTGTCCTGAGCATGGTTGACGAAGATGTCGTCTGTGTAGTCATGACTGAAGTCGAGATTGAGGAGAGCGATGTCACAGAT   1107
Sc-ia       CTATCCTGAGCATGGTTGACGAAGATGTCGTCTGTGTAGTCATGACTGAAGTCGAGATTGAGGAGAGCGATGTCACAGAT   1098
Sc-ib1/ib2  CTGTCCTGAGCATGGTTGACGAAGATGTCGTCTGTGTAGTCATGACTGAAGTCGAGATTGAGGAGAGCGATGTCACAGAT   1098
                             P8
Sc-j        TTCGACGATGAGGGCAACAAGCTCAAGTTCAAGGCCCAGTACGTTCTTGACATCGACGTGCGGCGTAAGCGGGTGTTATC   1187
Sc-ia       TTCGACGATGAGGGCAACAAGCTCAAGTTCAAGGCCCAGTACGTTCTTGACATCGACGTGCGGCGTAAGCGGGTGTTATC   1178
Sc-ib1/ib2  TTCGACGATGAGGGCAACAAGCTCAAGTTCAAGGCCCAGTATGTTCTTGACATCGACGTGCGGCGTAAGCGGGTGTTATC   1178
                                                                                   P7
Sc-j        CATCACCCAGCACCACATTGAAAGTATGGGTGATCTTATACCAGATCTCATCGCCTGTGAGTTCACTGCATACTCGGAGC   1267
Sc-ia       CATCACCCAGCACCACATTGAAAGTATGGGTGATCTTATACCAGATCTCATCGCCTGTGAGTTCACTGCATACTCGGAGC   1258
Sc-ib1/ib2  CATCACCCAGCACCACATTGAAAGTATGGGTGATCTTATACCAGATCTCATCGCCTGTGAGTTCACTGCATACTTGGAGC   1258
                                     P3                                     Stop codon
Sc-j        TCTCAAAAGGCATGCAGGCAATGGTGGAAGGAAATGAGGGTGAGGAAAGCACAAAGCGGATGAAGGTTAAATGATCTCTG   1347
Sc-ia       TCTCAAAAGGCATGCAGGCAATGGTGGAAGGAAATGAGGGTGAGGAAAGCACAAAGCGGATGAAGGTTAAATGATCTCTG   1338
Sc-ib1/ib2  TCTCAAAAGGCATGCAGGCAATGGTGGAAGGAAATGAGGGTGAGGAAAGCACAAAGCGGATGAAGGTTAAATGATCTCTG   1338

Sc-j        CATCATGTGGAACTTCGATGGTTTTTGCAACATATCAAGTAGGATTAACATTGAAGATTGGTGTTTGATTAGCACCCATC   1427
Sc-ia       CATCATGTGGAACTTCGATGGTTTTTGCAACATATCAAGTAGGATTAACATTGAAGATTGGTGTTTGATTAGCACCCATC   1418
Sc-ib1/ib2  CATCATGTGGAACTTCGATGGTTTTTGCAACATATCAAGTAGGATTAACATTGAAGATTGGTGTTTGATTAGCACCCATC   1418

Sc-j        TATGTGGAAGGATGAATGTGATGTAATCATTCTCTAGAAGTTATGATGTTATTATCAGATGGTAGTAAAACTGAATTGCT   1507
Sc-ia       TATGTGGAAGGATGAATGTGATGTAATCATTCTCTAGAAGTTATGATGTTATTATCAGATGGTAGTAAAACTGAATTGCT   1498
Sc-ib1/ib2  TATGTGGAAGGATGAATGTGATGTAATCATTCTCTAGAAGTTATGATGTTATTATCAGATGGTAGTAAAACTGAATTGCT   1498

Sc-j        AGTGTGTTTTGTTAACGCTGATGGTTTTAACATTACTTCATAGCTACCTTTGAATTGTCTGGTCATGCCGGGCTGGTTGA   1587
Sc-ia       AGTGTGTTTTGTTAACGCTGATGGTTTTAACATTACTTCATAGCTACCTTTGAATTGTCTGGTCATGCCGGGCTGGTTGA   1578
Sc-ib1/ib2  AGTGTGTTTTGTTAACGCTGATGGTTTTAACATTACTTCATAGCTACCTTTGAATTGTCTGGTCATGCCGGGCTGGTTGA   1578

Sc-j        TGGCTGACTCCGATAAGTGATACAAGTGGTACCTGATGATATCTTTTGTCAAACTCTAGC   1647
Sc-ia       TGGCTGACTCCGATAAGTGATACAAGTGGTACCTGATGATATCTTTTGTCAAACTCTAGC   1638
Sc-ib1/ib2  TGGCTGACTCCGATAAGTGATACAAGTGGTACCTAATGATATCTTTTGTCAAACTCTAGC   1638
```

Supplementary Figure 3. Alignment of the coding and UTR sequences of the *Sc* alleles. The sequences of 5' untranslation regions (UTRs), coding regions, and 3' UTRs of *Sc-j, Sc-ia*, and *Sc-ib1/Sc-ib2*, which were determined by sequencing the genomic DNAs and cDNAs of T65, E5, 93-11, and MH63 (and the public sequence of Nip). 93-11 and MH63 have the same sequences as *Sc-ib1/ib2* of E5 but lack *Sc-ia*. The primers (P#) used for various analyses are shown.

```
Sc-j   MAPTWVLLDRFVKPTIFDEEESKGKGESTGAPVKYLPARLRQEVPAGMRDVKPYPEVADP   60
Sc-ia  MAPAWVLLDRVVKPAVFDEEESKGKGESTGAPVKYLPARLRQEVPAGMRDVKPYPEVADP   60
Sc-ib  MAPAWVLLDRVVKPAVFDEEESKGKGESTGAPVKYLPARLRQEVPAGMRDVKPYPEVADP   60

Sc-j   PIISRFSMLISRKAIRVVKSVRVRCADKSLVLFYAGTGFPGFSSHGCHLIYDAIDGSLTA  120
Sc-ia  PIVSRFSMLISRKAIRVVKSIHVECADKSLVLFYAGTGFPGFS-HGCHLIYDAIDGSLTA  119
Sc-ib  PIVSRFSMLISRKAIRVVESIHVECADKSLVLFYAGTGFPGFS-HGCHLIYDAIDGSLTA  119

Sc-j   VHTFPFPVSGVVWVGRAAVLRHAGGGGGGGDGTTASYVIAELLRPFHGSLPDATLVMWLS  180
Sc-ia  VHTFPFPVSGVVWVGTAAVLRHAGGGGGG-DGTTA-YVIAELLRPFHGSLPDATLVMWLS  177
Sc-ib  VHTFPFPVSGVVWVGTAAVLRHAGGGGGG-DGTTA-YVIAELLRPFHGSLPDATLVMWLS  177

Sc-j   NSPASTSGSNGQWVKEDVRLPGEVCTGTDPFTTDLVFSFGESCLCWADLFMGILFCDLAT  240
Sc-ia  NSPASTSGSNGQWVKEDVRLPGEVCTGTDPFTTDLVFSFGESCLCWADLFMGILFCDLAT  237
Sc-ib  NSPASTSGSNGQWVKEDVRLPGEVCTGTDPFTTDLVFSFGESCLCWADLFMGILFCDLAT  237

Sc-j   LRAPRFRFIPLPKACSFDPVGKYGRPHMPEFRSMGRVNGVIRLIDMEGFTNEYLAVDEVK  300
Sc-ia  LRAPRFRFIPLPKACSFDPVGKYGRPHMPEFRSMGRVNGVIRLIDMEGFTNEYLAVDEVK  297
Sc-ib  LRAPRFRFIPLPKACSFDPVGKYGRPHMPEFRSMGRVNGVIRLIDMEGFTNEYLAVDEVK  297

Sc-j   LTIWTLSADLSEWEKGPVCTVGDIWASEEFVAMGLPQLRPMCPVLSMVDEDVVCVVMTEV  360
Sc-ia  LTIWTLSADLSEWEKGPVCTVGDIWASEEFVAMGLPQLRPMCPILSMVDEDVVCVVMTEV  357
Sc-ib  LTIWTLSADLSEWEKGPVCTVGDIWASEEFVAMGLPQLRPMCPVLSMVDEDVVCVVMTEV  357

Sc-j   EIEESDVTDFDDEGNKLKFKAQYVLDIDVRRKRVLSITQHHIESMGDLIPDLIACEFTAY  420
Sc-ia  EIEESDVTDFDDEGNKLKFKAQYVLDIDVRRKRVLSITQHHIESMGDLIPDLIACEFTAY  417
Sc-ib  EIEESDVTDFDDEGNKLKFKAQYVLDIDVRRKRVLSITQHHIESMGDLIPDLIACEFTAY  417

Sc-j   SELSKGMQAMVEGNEGEESTKRMKVK                                    446
Sc-ia  SELSKGMQAMVEGNEGEESTKRMKVK                                    443
Sc-ib  LELSKGMQAMVEGNEGEESTKRMKVK                                    443
```

Supplementary Figure 4. Alignment of the protein sequences encoded by *Sc-j* and the *Sc-i* paralogs. The putative DUF1618 domain is boxed. The amino acid variations among Sc-j, Sc-ia and Sc-ib (Sc-ib1and Sc-ib2) are shown in grey shading, and three amino acids of Sc-ib that differ from Sc-ia are shown in red. The estimated molecular weights of Sc-j and Sc-i are 48.9 kDa and 48.8 kDa, respectively.

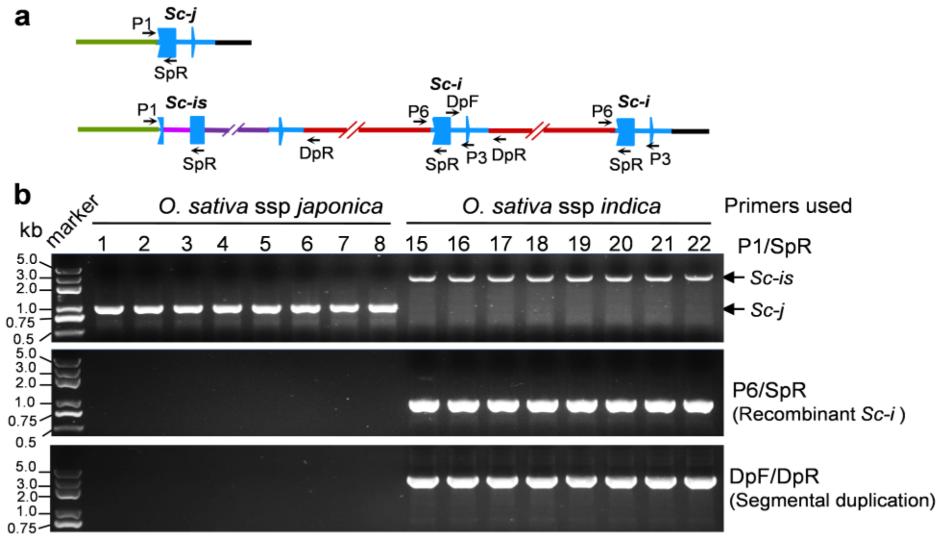

c

Code	Variety (acces.)	Habitat	Ssp.	Allele	Code	Variety (acces.)	Habitat	Ssp.	Allele
1	T65	Taiwan	*j*	*Sc-j*	19	93-11	EC	*i*	*Sc-i*
2	Zhonghua 11	NC	*j*	*Sc-j*	20	MH63	SC	*i*	*Sc-i*
3	Nipponbare	Jp	*j*	*Sc-j*	21	Chigu 2	SC	*i*	*Sc-i*
4	Houjiu	SC	*j*	*Sc-j*	22	Xiangzhan	SC	*i*	*Sc-i*
5	Zhaojing 2	EC	*j*	*Sc-j*	23	Tiejiaozhan	CC	*i*	*Sc-i*
6	Heimangjing	SC	*j*	*Sc-j*	24	Daluozhan	SC	*i*	*Sc-i*
7	Yamagata 97	Jp	*j*	*Sc-j*	25	Shazhan	SC	*i*	*Sc-i*
8	Fukukei 8127	Jp	*j*	*Sc-j*	26	Eshandabaigu	SWC	*i*	*Sc-i*
9	Higaxi 1076	Jp	*j*	*Sc-j*	27	Shuzhouzhan	EC	*i*	*Sc-i*
10	Hujikei 209	Jp	*j*	*Sc-j*	28	Sichuanzhan	SWC	*i*	*Sc-i*
11	Zhonghua 18	NC	*j*	*Sc-j*	29	Baizhan	SWC	*i*	*Sc-i*
12	Xiangjing 227	NC	*j*	*Sc-j*	30	Xizhan	SC	*i*	*Sc-i*
13	Liaoxing 15	NEC	*j*	*Sc-j*	31	Baizhan 81	SC	*i*	*Sc-i*
14	Zhongguo 177	NC	*j*	*Sc-j*	32	Baihuazhao	SC	*i*	*Sc-i*
15	E5 (T65-NIL)		NIL	*Sc-i*	33	Liushirizhao	SC	*i*	*Sc-i*
16	HHZ	SC	*i*	*Sc-i*	34	Maohe	SC	*i*	*Sc-i*
17	ZS97	SC	*i*	*Sc-i*	35	Guangzhan	SC	*i*	*Sc-i*
18	GLA4	SC	*i*	*Sc-i*	36	Kaoanmoya	SC	*i*	*Sc-i*

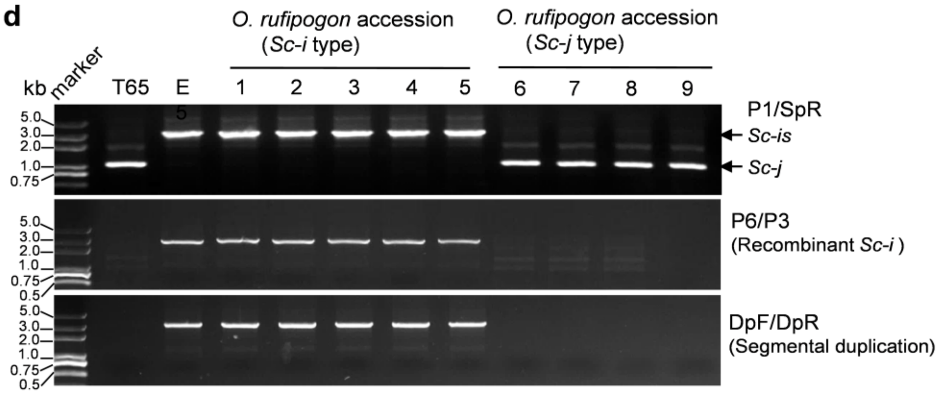

Supplementary Figure 5. Detection of the structural variation and tandem duplication in the cultivated and wild rice. (**a**) Locations of the PCR primers in the *Sc-j* type and *Sc-i* type structures used in this analysis. (**b**) Examples of genotyping the *Sc*

locus in *japonica* and *indica* varieties (lines). The code numbers are as shown in (**c**). Note that this PCR assay could determine the presence of the tandem segmental duplication, but could not estimate the actual copy numbers of the duplicated segment (e.g., at least two copies of *Sc-i*). (**c**) The *Sc* genotypes in the analyzed *japonica* (*j*) and *indica* (*i*) cultivars (lines). Jp, Japan; NC, North China; SC, South China; EC, East China; NEC, North-east China; CC, Center China; SWC, South-East China; Ssp, Subspecies. (**d**) Genotyping of the *Sc* locus in *O. rufipogon* accessions. *Sc-j* type and *Sc-i* type alleles were detected in different accessions.

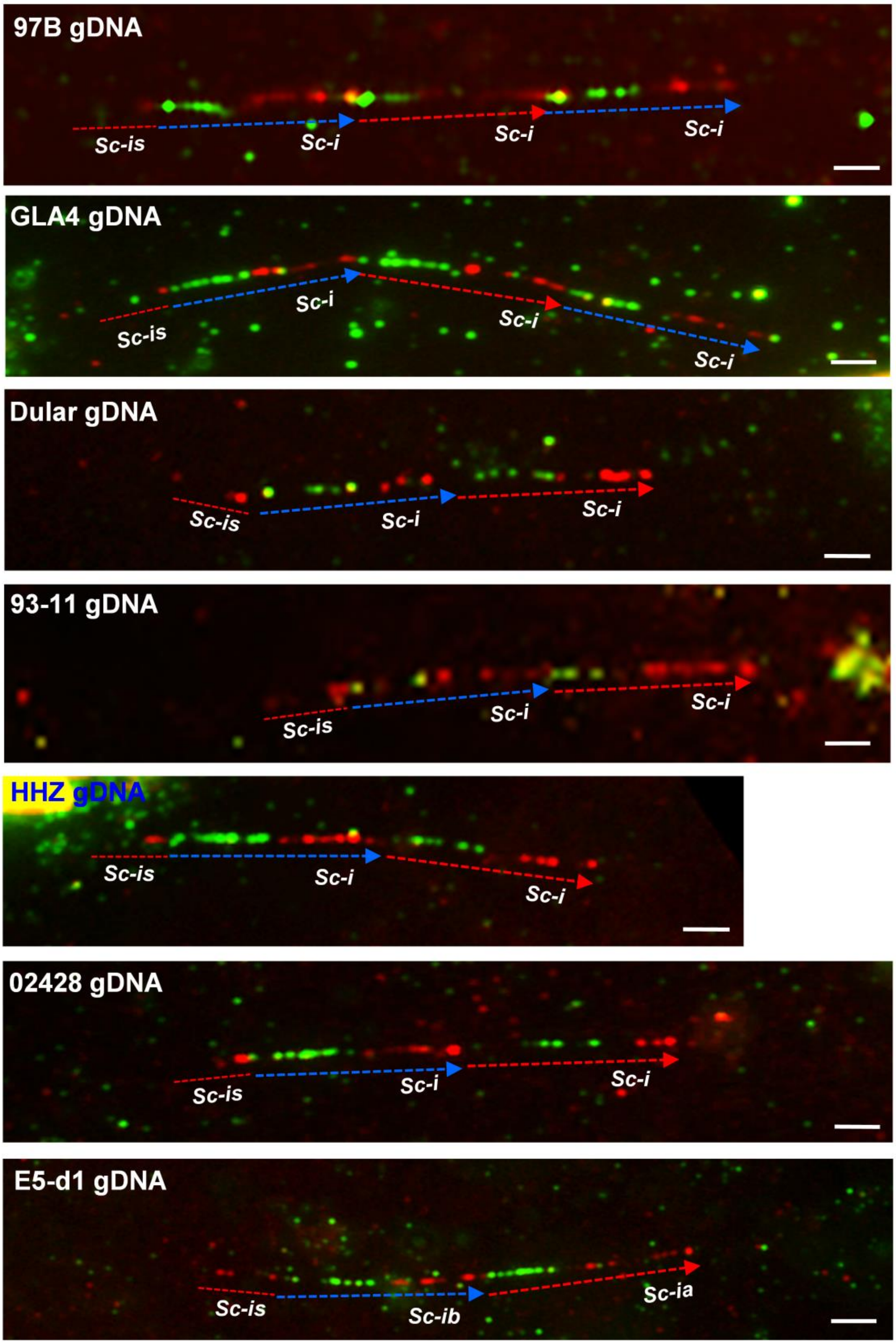

Supplementary Figure 6. Determination of *Sc-i*-containing segmental duplications in rice by genomic DNA fiber-FISH. Probes I and II (see **Fig. 1d**) were labeled, as did for E5 gDNA (**Fig. 1d**), for detection with red and green fluorescence, respectively, and mixed for hybridization with genomic DNA of *indica* cultivars and the targeted-deletion plant E5-d1 (in which one of the three ~28-kb segments was deleted, see **Fig. 4a**). Scale bars, 2 μm.

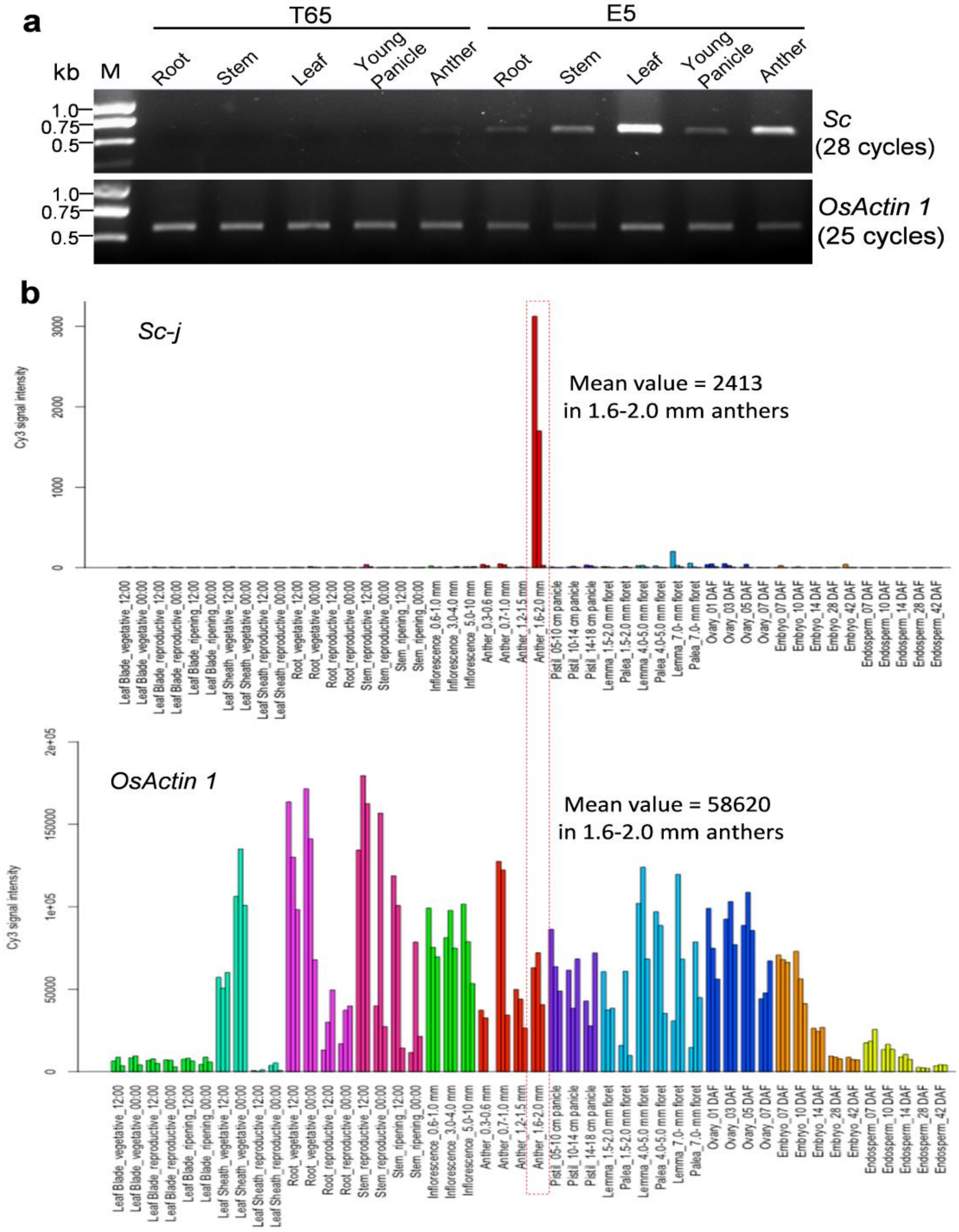

Supplementary Figure 7. Expression analysis of the *Sc* gene. (**a**) Semi-qRT-PCR of *Sc* gene expression in various tissues of T65 and E5. The anthers are at the late microspore to bicellular pollen stages. (**b**) Expression profiles of *Sc-j* (Os03g0247300) and the reference gene *OsActin 1* (Os03g0718100) in the *japonica* cultivar Nip, based on the Rice Expression Profile Database (RiceXPro, http://ricexpro.dna.affrc.go.jp/)[50]. The anthers of 1.6-2.0 mm long are at the bicellular pollen stage; anthers of this stage consists of pollen grains and anther wall (epidermis) cells, while the tapetum and middle layer are degenerated completely. The relative expression level of *Sc-j* to *OsActin 1* is 0.041 (2413/58620).

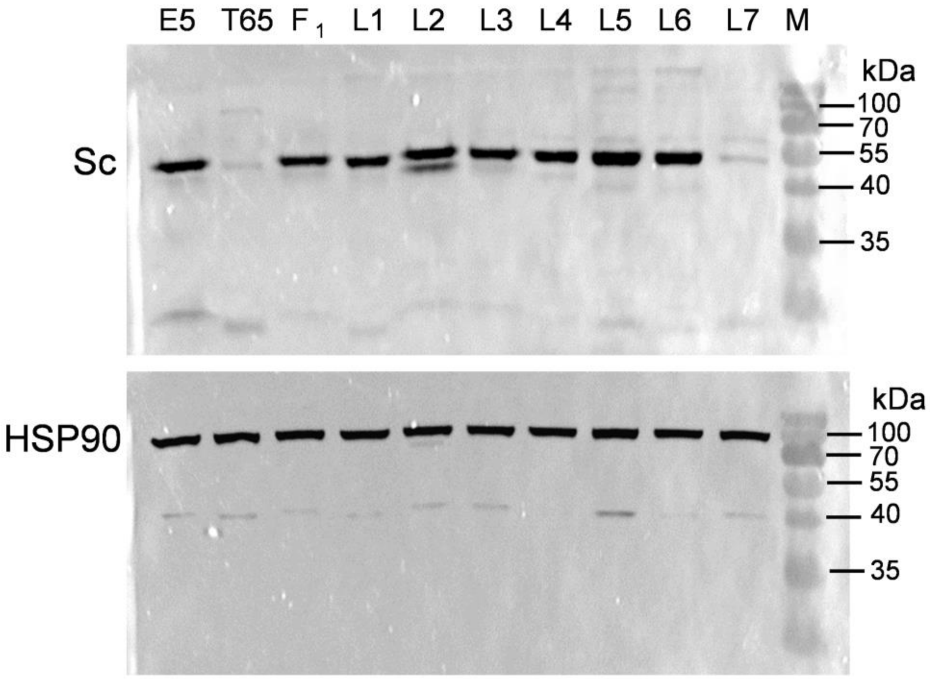

Supplementary Figure 8. Protein blotting analysis of Sc in rice lines. The Sc proteins (48.8 kDa and 48.9 kDa) were blotted and detected with anti-Sc antibodies from protein extracts of anthers (at stages of uninucleate late-microspore to early-bicellular pollen) of E5, T65 and the F_1, and other rice lines carrying *Sc-i* (L1-L6) and *Sc-j* (L7). Detection of HSP90 (heat shock protein 90, 90 kDa) served as the protein loading control. M, molecular weight marker.

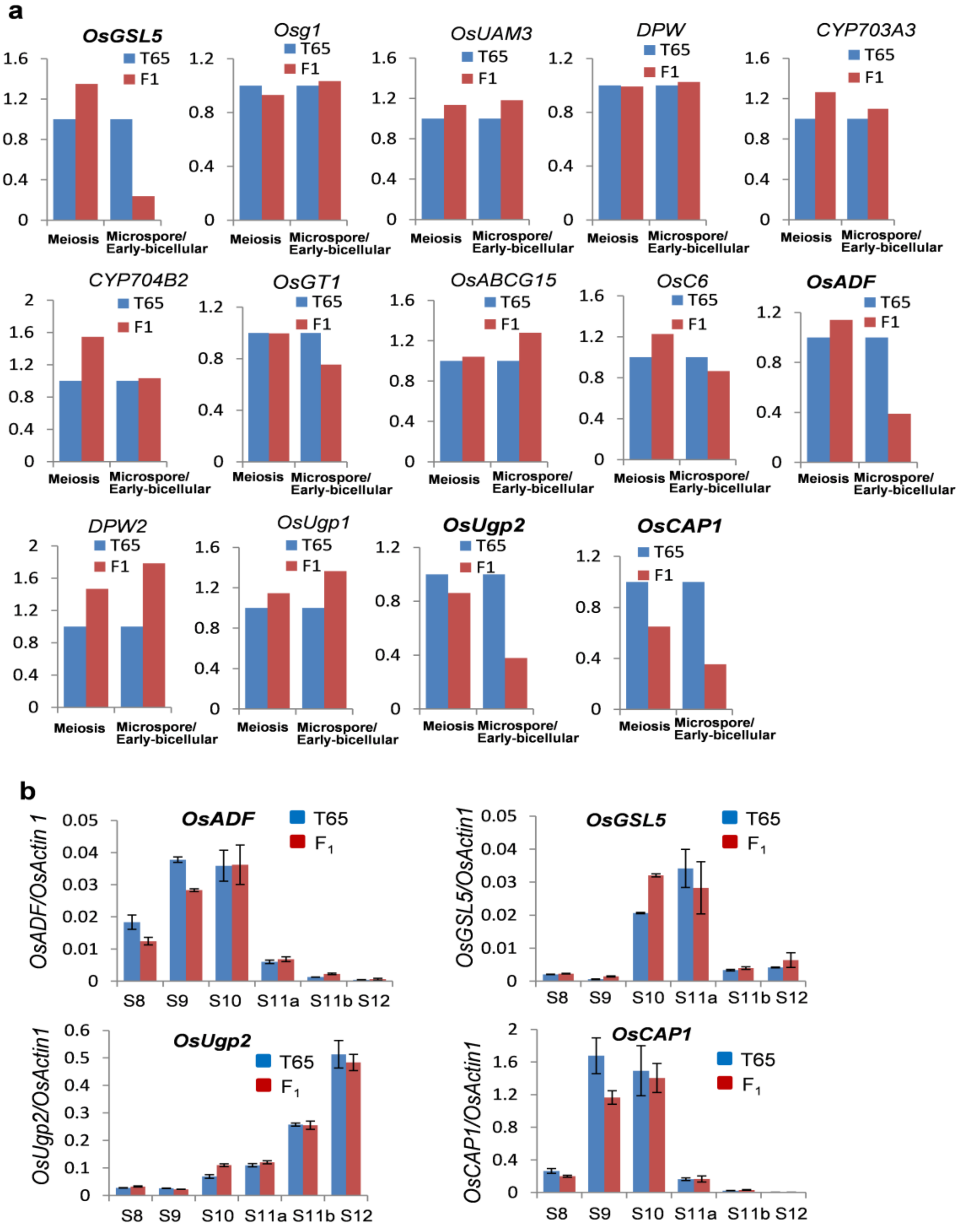

Supplementary Figure 9. Expression analysis of genes known for microspore development in anthers of the F_1 and T65. (**a**) Relative expression levels of the F_1 (T65/E5 cross) to T65 of 14 genes based on an Affymetrix Microarray transcriptome data set from RNAs of anthers at meiosis and microspore/early-bicellular stages. (**b**) qRT-PCR analysis of four genes in developing anthers of the F_1 and T65. S8 to S12 indicate anther samples at stages of meiocyte/meiosis, early-microspore, late-microspore, early-bicellular pollen, late-bicellular pollen, and tricellular pollen, respectively. Data are shown as means ± s.e.m. (n = 3).

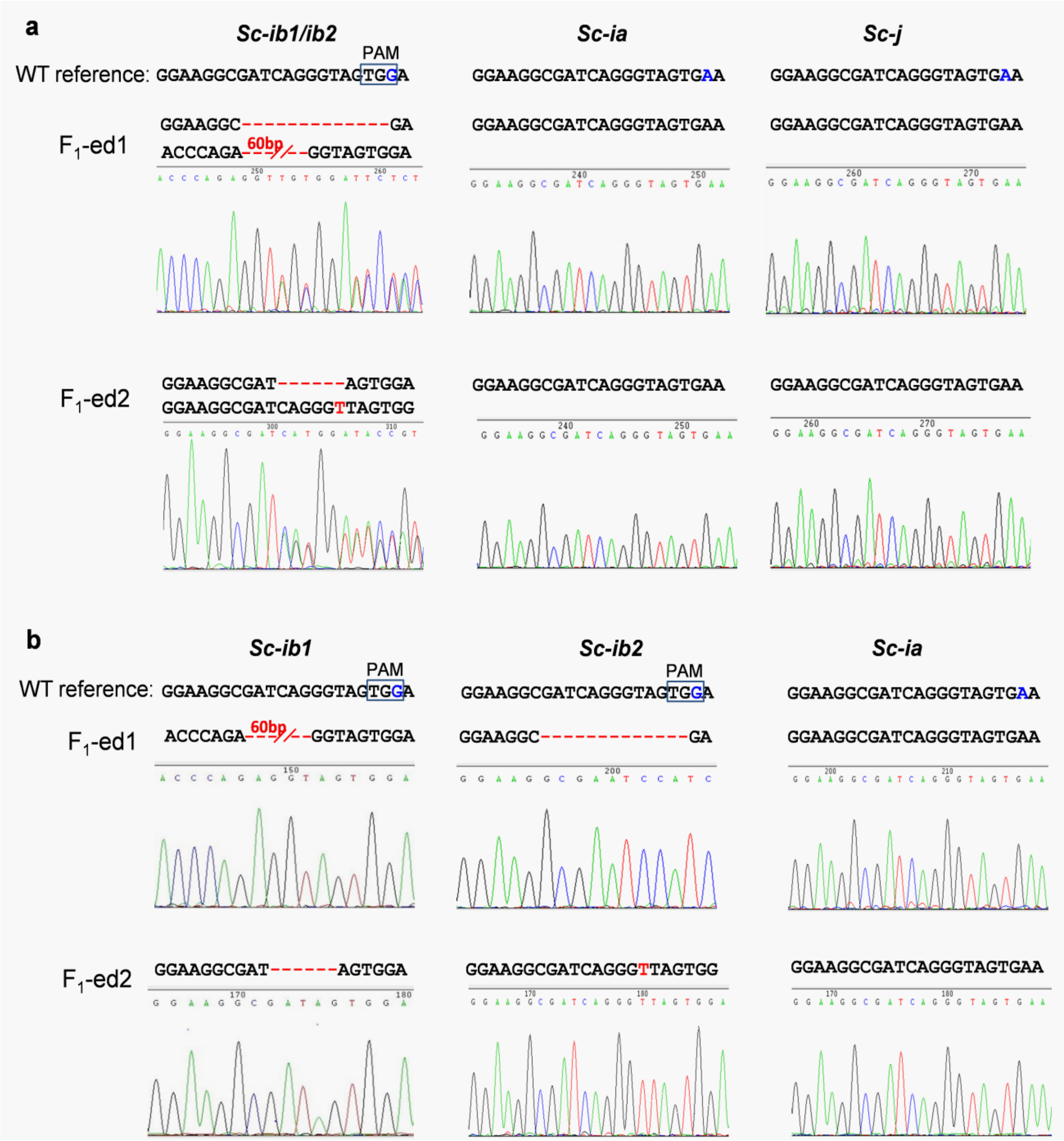

Supplementary Figure 10. Sequencing of PCR amplicons containing the CRISPR/ Cas9-editing sites. (**a**) The PCR amplicons with the editing site in *Sc-ib1/Sc-ib2* and the corresponding site in *Sc-ia* and *Sc-j* from F_1-ed1 and F_1-ed2 were directed sequenced, and the superimposed sequencing chromatograms were decoded with the web-tool DSDecode (http://dsdecode.scgene.com/; http://skl.scau.edu.cn)[52,53]. (**b**) The amplicons with the sites in *Sc-ib1/Sc-ib2* and *Sc-ia* were cloned into a plasmid vector and several clones were sequenced.

Supplementary Tables 1-3

Supplementary Table 1. The gene copy numbers and relative transcript levels of *Sc-j, Sc-ia,* and *Sc-ib1/ib2* (*Sc-ib*), and copies in E5 and the F_1.

Sample	No. clone	No. clones with *Sc-j*:*Sc-ia*:*Sc-ib*	% of *Sc-j* clones
E5 gDNA	196	0:58:138	-
F_1 gDNA	270	71:63:136	26.3
F_1 cDNA (S8)	363	0:135:228	-
F_1 cDNA (S9)	286	2:106:178	6.9
F_1 cDNA (S10)	380	3:128:253	7.9
F_1 cDNA (S811a)	380	18:109:253	4.7

Note: a fragment of the *Sc* genomic DNA (gDNA) and cDNA of each anther sample, which included all the alleles (paralogs) sequences, was amplified using primers P5 and P7 (Supplementary Fig. 3), and cloned into a plasmid vector. Then the clones were genotyped based on the polymorphic markers among *Sc-j* and the *Sc-i* paralogs. S8 to S11a indicate anther samples at stages of meiocyte/meiosis, (uninucleate) early-microspore, (uninucleate) late-microspore, and early-bicellular pollen, respectively. The ratios of the gDNA clones of *Sc-ia* and *Sc-ib* (*Sc-ib1*, *Sc-ib2*) and from E5 fit 1:2 ($P >$ 0.05), and those of *Sc-j, Sc-ia* and *Sc-ib* from F_1 fit the 1:2:1 ratio ($P > 0.05$), confirming that there are one copy *Sc-ia* and two identical copies of *Sc-ib1* and *Sc-ib2* in E5, and further indicating that no significant amplification bias occurred among the *Sc-j* and *Sc-i* paralog sequences during PCR. The approximate 1:2 ratio of the total *Sc-ia*:*Sc-ib1/ib2* cDNA clones (478:912) suggests that the three *Sc-i* paralogs were expressed at approximately the same levels.

Supplementary Table 2. Male phenotype and segregation analysis of *Sc-j*-antisense transgenic lines.

Transgenic line family	No. T-DNA site	Phenotype In T_0	No. T_1 plants	Segregation in T_1 (T+: T-)	χ^2 (3:1)	χ^2 (1:1)
Anti-1	1	SS	110	54:56	39.38**	0.04
Anti-2	1	SS	50	29:21	7.71**	0.32
Anti-3	1	SS	29	12:17	17.48**	0.86
Anti-4	1	SS	37	21:16	6.57*	0.68
Anti-5	1	SS	37	22:15	4.77	1.32

Note: SS, semi sterility. T+ and T- represent the presence (in a hemizygous state) and absence of the T-DNA (containing the antisense transgene) in the segregants of the T_1 families, respectively. * and ** represent significance at $P < 0.05$ and $P < 0.01$ for the chi-square test, respectively.

Supplementary Table 3. Primer sequences used in this study.

Primer	Primer sequence (5' - 3')	Primer sequence (5' - 3')	Purpose
P24-38	CAGTTTGGAAGCTGATGCAG	CCAAGGAGCAACAACTCAAC	Marker 38 (1st PCR) for mapping
P24-38 I	GAGCCTGCAGCTACGGATAA	CCAAGGAGCAACAACTCAAC	Marker 38 (*indica*-specific SNP)
P24-38 J	TTTTTGAGCCTGCAGCTACGGATGC	CCAAGGAGCAACAACTCAAC	Marker 38 (*japonica*-specific SNP)
P24-80.4	GCGCCTGCAGATCTGTAGTG	CTACTCGACGTGTGCCGTTC	Mapping
P24-84.4	ACAAAGCAAATAGGAGAT	CGTGGCCGCACTTGCTAC	Mapping
P24-85.7	CAGGCTCGATATCAGCATAG	GTATAGAGCGTGTGCTACAG	Mapping and segregation analysis
P24-90.5	CCACCCAGGCGCAA	CTTCACGTCCCGCATGCC	Mapping
P24-92.0	CATTGGTTAAAGGATGGTAC	TAGTACTGCTAGGACCATCC	Mapping
P24-93.0	GCTTCGCTCCCGTCATCATC	ACAACTCCAGTAAGAACTCC	Mapping
P24-100.7	TAGGGTTTGGGGAGCTGAGG	GGATGACGACCGGAGATAGG	Mapping
P-Sc-j	ATCGGGAGCGGAACATCCATC	TTCCTGCAAAGCTAACCTTGGG	PCR for sequencing *Sc-j*
P-Sc-is-1	CCCTGTCTTGCGCACGGTCAAC	ACCCCATGGCGACGAATTCC	PCR for sequencing *Sc-is*
P-Sc-is-2	GTTTCATCCCATTGCCCAAG	CACGTTCTCGCGGCAACGTAC	PCR for sequencing *Sc-is*
P-Sc-is-3	GTACGTTGCCGCGAGAACGTG	ACTGACGAAACAAATGATCGC	PCR for sequencing *Sc-is*
IRS-1	ACGTTTAGATCATCATAACTCCATC	GTAGTCGTATTGGACTCTATCAGG	PCR for sequencing the 23.9-kb
IRS-2	CCTGATAGAGTCCAATACGACTAC	CCATAAAGAATCTCTGTGCACTG	PCR for sequencing the 23.9-kb
IRS-3	GGTCCATGATGCACCTAGTC	GTGGGCTGAAGAATTACAC	PCR for sequencing the 23.9-kb
IRS-4	TGTGTAATTCTTCAGCCCAC	CACCAGTATCAAGAATGGAC	PCR for sequencing the 23.9-kb
IRS-5	GTCCATTCTTGATACTGGTG	ACCCCATGGCGACGAATTCC	PCR for sequencing the 23.9-kb
P-Sc-ib	ATGGCTCCCACCTGGGTCTTG	TTGCTCATGCTCTGCGAACC	PCR for sequencing *Sc-ib1/ Sc-ib2*
P-Sc-ia	ATGGCTCCCACCTGGGTCTTG	GAACTAAAGCAAACTCATGTG	PCR for sequencing *Sc-ib1/ Sc-ia*
Actin1qRT	CATCTCTCAGCACATTCCAG	GAACCACAGGTAGCAATAGG	qRT-PCR
P8/P3	CAACAAGCTCAAGTTCAAGG	TCACCCTCATTTCCTTCCAC	qRT-PCR in Fig. 2a
P1/P2	AGGCGCAAATCATCCGCC	ATCCGCAACCTCTGGGTAG	*Sc-j*-specific primers for qRT-PCR
P9a/P10	GAAGGCGATCAGGGTAGT	TGCACTGCAGTGAGAGAA	qRT-PCR for *Sc-ia* of edited plants and three *Sc-i* copies of F_1 plants
P9b/P10	AAGCCCTACCCAGAGGTA	TGCACTGCAGTGAGAGAA	qRT-PCR for Sc-ib/ed1 mutated *Sc-i*
P9c/P10	GATTTCGCGGAAGGCGAA	TGCACTGCAGTGAGAGAA	qRT-PCR for Sc-ib/ed1 mutated *Sc-i*
P9d/P10	TTCGCGGAAGGCGATAGT	TGCACTGCAGTGAGAGAA	qRT-PCR for Sc-ib/ed2 mutated *Sc-i*
P9e/P10	GAAGGCGATCAGGGTTAG	TGCACTGCAGTGAGAGAA	qRT-PCR for Sc-ib/ed2 mutated *Sc-i*
Actin1SqRT	CTGATGGACAGGTTATCACC	GAACCACAGGTAGCAATAGG	Semi-qRT-PCR
P4/P3	GTTTCATCCCATTGCCCAAG	TCACCCTCATTTCCTTCCAC	Semi-qRT-PCR
OsADF	GGAGAAGCTGACATTGAAAC	CGTAGCCGTCCAGGTGGTTG	qRT-PCR
OsGSL5	GTTGGATTTTATATCAGTTC	CCTAGCTGCACAATAGATTG	qRT-PCR
OsUgp2	GAGTTCAAGAAGGTCGGGTG	CCAGTTTGACACCAGGTTG	qRT-PCR

OsCAP1	GCTTGAAAAGCAGTGAAGAG	GACAAATGTTTGTGTGGACC	qRT-PCR
Actin1-RT	GATGTTATTCATTTCATAGA		Primer for reverse transcription
Sc-RT	TCATTTAACCTTCATCCGC		Primer for reverse transcription
Dp	GTATCCAATTGAATTCTTGATTCAG	GTAACTTTTGTGGATAGATGGAG	Duplication detection
P1/Sp6R	AGGCGCAAATCATCCGCC	ACCCCATGGCGACGAATTCC	Genotyping
P6/P3	GTTCTTGCCGTCTCTTGCAC	TCACCCTCATTTCCTTCCAC	Genotyping
Sc-j-EN	TTTGTCGATGCTCACCCTG	GGTTGCCGCAGCTTAGAC	Segregation analysis
Sc-j-EX	CCTTGAACTTGAGCTTGTTG	GGTTGCCGCAGCTTAGAC	Segregation analysis
Hpt	GGACGATTGCGTCGCATCGACCCTG	CTCGTGCTTTCAGCTTCGATGTAGG	Transgene analysis
P5/P7	AAGGTAAGGGAGAATCGACC	CTGCATGCCTTTTGAGAGCTCC	Genotyping in f ig. 9
3x3-bp Indel	CACATCACGAGCGTGGCATC	TGTGTCCGGAGTCGTCTGGG	Indel marker for *Sc-j* and *Sc-i* paralogs
SNP	CAGGGTAGTGGAATCC	CTCTTGTCGGCGCAC	SNP marker for *Sc-ia* and *Sc-ib*
Probe1-1	GGTCCATGATGCACCTAGTC	GTGGGCTGAAGAATTACAC	Fiber-FISH
Probe1-2	TGTGTAATTCTTCAGCCCAC	CACCAGTATCAAGAATGGAC	Fiber-FISH
Probe1-3	GTCCATTCTTGATACTGGTG	ACGTAGAGACGACGCATG	Fiber-FISH
Probe2-1	ACGTTTAGATCATCATAACTCCATC	GTAGTCGTATTGGACTCTATCAGG	Fiber-FISH
Probe2-2	CCTGATAGAGTCCAATACGACTAC	CCATAAAGAATCTCTGTGCACTG	Fiber-FISH
Sc-ia/b-D （U3）	GGCAGGCACAATGAGCGGGCTGGA	AAACTCCAGCCCGCTCATTGTGCC	CRISPR construct for duplication deletion
Sc-ia/b-D （U6a）	GCCGCCAATGGGTTCCTGACACAT	AAACATGTGTCAGGAACCCATTGG	CRISPR construct for duplication deletion
Sc-ib-ED	GCCGGCGGAAGGCGATCAGGGTAG	AAACCTACCCTGATCGCCTTCCGC	CRISPR construct for editing *Sc-ib1/2*
Anti-*Sc-j*	CCGCCAAGCTTGATGCCACGCTCGT GATGTG	TGAAAGGTACCCTGCATGCCTTTTGA GAGCTCC	Antisense construct
